# Neurocysticercosis: A Rare Cause of Headache Needing Craniotomy

**DOI:** 10.31480/2330-4871/154

**Published:** 2022-03-08

**Authors:** Xiaowei Lu, Ning Miao, Andrew Mannes

**Affiliations:** Department of Perioperative Medicine Clinical Center, National Institute of Health, USA

## Introduction

Cysticercosis is a parasitic infection caused by larval of the pork tapeworm (Taenia solium). In humans, the larval cysts can infect body tissue including muscle, skin, eyes, and central nervous system (CNS). Larval cysts in the CNS cause a form of cysticercosis called neurocysticercosis (NCC), a leading cause of seizures and epilepsy [1,2] which is fatal if left untreated.

The tapeworm is endemic in most developing countries where pigs are raised, including Latin American, sub-Saharan Africa, and large regions of Asia. The human can act as both intermediate and definitive host acquiring the tapeworm after ingesting infected pigs with cysticerci or larvae in its tissue or water contaminated with human feces. Cysticerci or larvae attach to the small intestine of humans by a scolex, or head, which extends into segments referred to as proglottids. Humans can also ingest food-containing ova that hatch into oncospheres that invade the blood vessels of the intestine and disseminate into subcutaneous tissue, muscle, and brain [1].

We report a case of a 30-year-old male with a history of chronic headaches (HA) since childhood and new onset generalized tonic-clonic seizures. He was diagnosed with neurocysticercosis and referred to our hospital for surgical resection of cysticercosis in the fourth ventricle.

## Case Presentation

**Figure 1:**
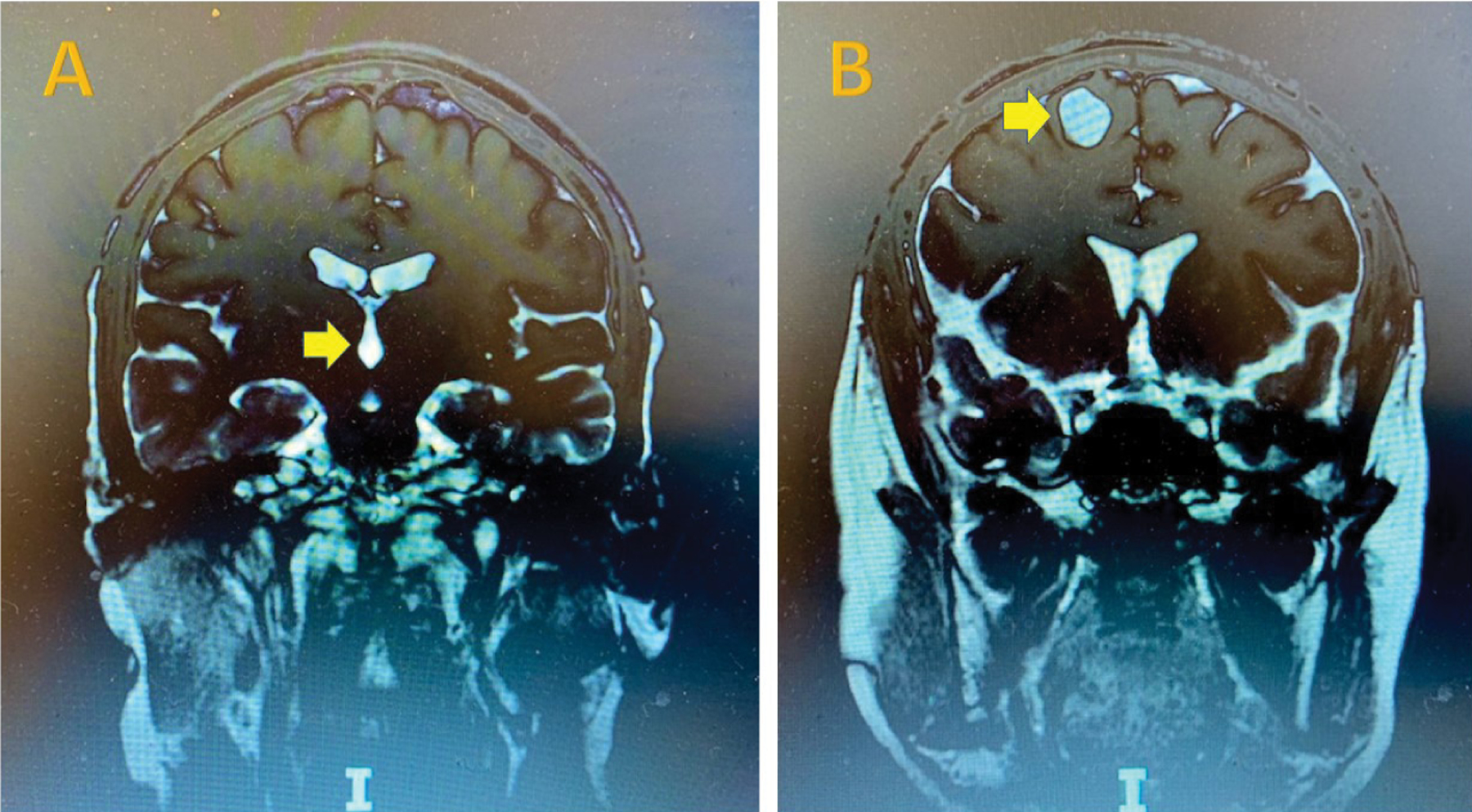
Cystic lesions on MRI. 4^th^ ventricle and right frontal lobe scoleces in cysts with arrows.

A thirty-year-old male patient from Honduras with a history of chronic HA was in his usual state of health until he experienced two generalized tonic-clonic seizures. He was treated with the anti-seizure medication levetiracetam and dexamethasone for epilepsy and presumed brain inflammation from NCC. He was subsequently referred to our hospital for further evaluation. The brain MRI revealed two major lesions: A viable parenchymal cyst lesion (14 × 17 × 20 mm) in the right frontal lobe with surrounding edema and an intraventricular cyst lesion within the fourth ventricle. There were also multiple calcified brain lesions in subarachnoid space (Figure 1). He was found to be positive for cysticercus antibodies but HIV, toxoplasma, strongyloides, histoplasma, cryptococcus and TB titers were all negative. His lab results, examination and imaging studies confirmed the patient’s diagnosis of neurocysticercosis.

The patient denied any other neurological symptoms (nausea, vomiting, weakness, loss of consciousness, or imbalance). Due to concerns of worsening inflammation/edema and obstructive hydrocephalus, anti-parasitic medications were not initiated, and a neurosurgeon was consulted for removal of intraventricular lesion. The epilepsy team also evaluated the patient to assess the benefit of the surgical resection of the cystic lesion on the right frontal lobe to eliminate his seizure activity. The team concluded that the patient’s seizures were well controlled and not intractable and, with the multiple other active/calcified lesions in the cortex, the risks outweighed the benefits in removing the right frontal lesion. Thus, the team would proceed with surgical removal of only the fourth ventricle lesion and then begin anti-parasitic therapy.

The patient was taken to operating room and ASA standard monitoring placed. The patient pre-oxygenated with 100% O_2_ and induced with IV fentanyl, propofol and rocuronium. An endotracheal tube was placed smoothly and anesthesia was maintained with air, O_2_, sevoflurane, fentanyl and intermittent rocuronium. An arterial line was inserted in the right radial artery and the bladder was catheterized.

**Figure 2:**
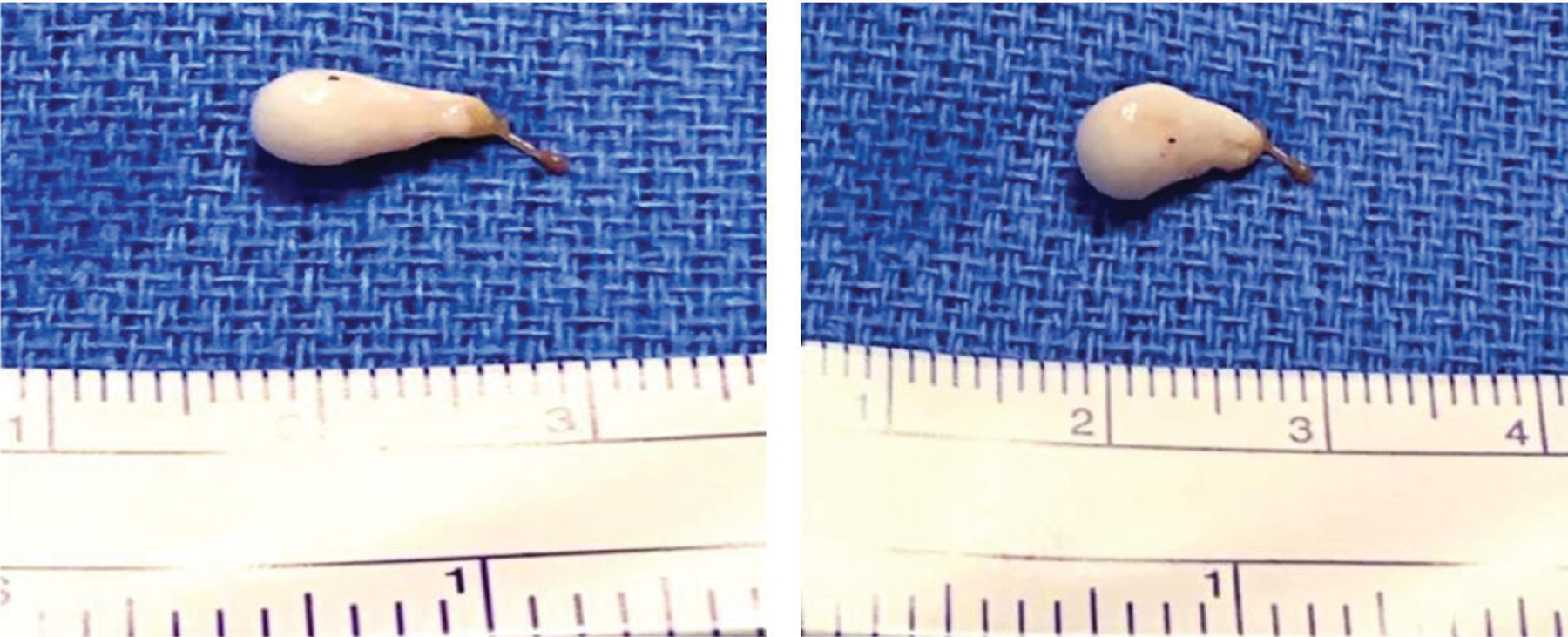
Surgical resection of one neurocysticercosis scolex and associated cyst in 4^th^ ventricle.

The surgeon resected the complete encapsulated cyst from within the fourth ventricle (Figure 2). No major intraoperative complications were observed and his postoperative course was uneventful. He was discharged from the hospital on day 12.

The patient returned for post-operative follow-up at 4 weeks and repeat MRI imaging showed the complete resection of the fourth ventricular cystic lesion and a size reduction of the right frontal cystic parenchymal lesion. The patient denied any interim change to health status and no headaches or seizures were reported since the procedure.

## Discussion

NCC is the most common neurologic parasitic infection of the central nervous system. The World Health Organization (WHO) estimates the total number of people suffering from NCC, including symptomatic and asymptomatic cases, to be between 2.56–8.30 million worldwide. NCC is typically considered a disease of the developing world, however, it has steadily increased in the developed nations, especially in the United States. This increase has largely been driven by the influx of immigrants from endemic to non-endemic regions [2–4]. US CDC reported that there are an estimated 1,000 new hospitalizations for NCC in the United States annually primarily in New York, California, Texas, Oregon, and Illinois. The mortality rate of NCC is estimated to be 0.06 per million people in the USA [3].

NCC cysts pass through three stages in the human brain: 1) Active vesicular cyst where the parasite is viable, 2) Degeneration of the parasite (colloidal, nodular/granular) and 3) Formation of calcified granulomas [2,5]. The most common symptoms and signs of NCC included seizures, hydrocephalus and headache. NCC should be suspected in high-risk patients with new onset headaches or seizures. Headache indicated the possible presence of hydrocephalus, meningitis or increased intracranial pressure. Some patients with NCC appeared to have cognitive deficits [2,4].

The diagnosis of NCC is confirmed based on the epidemiologic history, clinical manifestations, the presence of positive CSF or serum Taenia solium antibody titers and by neuroimaging (CT and MRI detection of brain parenchymal or extra-parenchymal scolex and cyst lesion with associated calcification and perilesional edema).

The primary treatments for NCC are anti-parasitic medications: Albendazole, praziquantel or a combination of both. The success rate of combination is higher than a single medication [6]. Worsening of the clinical symptoms can occur with anti-parasitic drugs, which can induce inflammatory reactions in the patient secondary to the destruction of the parasite and occlusion of the vessel around the cyst. Patients, who develop hydrocephalus resulting from cyst migration or destruction, have a high mortality rate. Corticosteroids (prednisone or dexamethasone) are prescribed to reduce the incidence of chronic cysticercosis arachnoiditis or encephalitis, and brain edema. Mannitol is used for acute intracranial hypertension secondary to NCC [3,7,8]. In the presence of hydrocephalus and increased intracranial pressure, anti-parasitic therapy is relatively contraindicated, unless a shunt is placed [2,4] or after the surgical removal of cysts to avoid ventricular obstruction.

Seizure is another clinical manifestation of NCC, occurring in 50–70% of NCC patients as the primary sign, with one report as high as 90% [9]. Multiple causes include direct inflammatory damage to the brain, gliosis, and genetic susceptibility [10]. Seizure activity increases with the presence of a vesicular cyst and during the degenerating stage, and it becomes less active with calcified granuloma. However one study suggested the calcified lesions as a possible source if recurrent seizure [11]. Anti-parasitic medications and surgical treatment of NCC are used to reduce the numbers of cysts and resulting frequency of seizures. Anticonvulsant therapy is the mainstay of managing NCC-associated seizure disorders, which usually responds well to first-line antiepileptics. Phenytoin, carbamazepine and levetiracetam are recommended but need to be guided by local availability, cost, drug interactions, and potential side effects [8].

Typically, NCC invasion of the subarachnoid space predominates, followed by the parenchymal, intraventricular and spinal forms. Ventricular system involvement of NCC usually causes ventricular dilatation and hydrocephalus that require surgical intervention. Studies have shown a strong association of seizure onset with the presence of active cysts/calcified lesions, especially multiple cysts with calcification in the frontal and temporal lobes. The recurrence rate of seizures with calcified lesions are much higher than that with lesion resolution [2,6,8,12]. Calcified NCC can cause brain inflammation from host immunologic response. Blood-brain barrier breakdown, cortical necrosis, space-occupying effect of NCC and reactive gliosis around calcified NCC can all lead to the chronic epileptogenic process [12,13]. The predictors of calcification usually include NCC lesion size > 10 mm, delayed treatment and inadequate dosage of medication [6]. Our patient reported long-term headaches since childhood and with recent onset of generalized grand-mal seizures. The MRI imaging was remarkable for one viable parenchymal cyst lesion (14 × 17 × 20 mm) in the right frontal lobe with surrounding edema, an intraventricular cyst lesion within the 4^th^ ventricle, and multiple calcified brain lesions.

The NCC patient with signs of intracranial hypertension (ICP), such as nausea, vomiting, papilledema, or altered mental status, should be aggressively managed preoperatively to avoid further increases. The placement of an arterial line allows continuous blood pressure monitoring, and maintenance of blood pH, use of moderate hyperventilation and administration of a hyperosmotic agent (e.g. mannitol or 3% normal saline) canoptimize cerebral perfusion and further decrease ICP.

Seizure management is an essential aspect of the anesthesia care of NCC patients. Oral dosing of anti-epilepsy medication (e.g. phenytoin, valproic acid, and phenobarbital) should be continued on the morning of the surgery regardless of NPO status but alternatively can be switched to the parenteral dosing if available. For prolonged surgical cases, these medications need to be re-dosed. The anesthesia provider should also closely monitor and correct hypoxia, hyper/hypoglycemia, and hypothermia which can impact the seizure threshold. However, there can be a balance between hyperventilation required to alleviate the ICP and the risk of hyperventilation as a trigger for inducing intraop seizures. Volatile agents can have a proconvulsant effect when used at higher than minimum alveolar concentrations (MAC). Sevoflurane has a highest seizure spike activity when compared to isoflurane and desflurane. Intravenous anesthesia agents such as propofol and midazolam suppress seizure activity, while etomidate, ketamine, and high dose of opioids can increase epileptogenic potential.

The pharmacologic effects of a neuromuscular blocker on an NCC patient are altered by the co-administration of drugs including anticonvulsants and steroids. Antiepileptic drugs generally cause resistance to non-depolarizing neuromuscular blockers and accelerate recovery from anesthesia. It is advisable to closely monitor the neuromuscular activity during surgery especially with the chronic use of older generation AEDs such as phenytoin and carbamazepine. However, the acute administration of phenytoin to a naive patient may potentiate the effect of neuromuscular blockade [14]. It has been previously reported that the long-term use of neuromuscular blockade together with steroid can cause myopathy, but the current studies on the correlation of muscle weakness in the short-term application of combined NMB and steroid do not show a clinically significant adverse effect [15].

## Conclusion

NCC is a leading cause of epilepsy in developing countries and is increasingly prevalent in the United States. NCC should be considered in the differential diagnosis in patients from endemic areas, with headache and epilepsy who have imaging consistent with calcified lesions and with scolex and cystic changes in subarachnoid, parenchymal and intraventricular space. Anesthesiologists should assess and document any preoperative pre-existing neurological deficit and intracranial hypertension (ICP) and the anesthetic plan should include the prevention of elevating intraoperative ICP (e.g. smooth emergence from anesthesia, avoiding stimulation of cough/hypertension and controlling postoperative pain and nausea/vomiting). Antiepileptic medication and steroids should be maintained throughout the perioperative period.
